# Too much of a good thing? An observational study of prolific authors

**DOI:** 10.7717/peerj.1154

**Published:** 2015-08-18

**Authors:** Elizabeth Wager, Sanjay Singhvi, Sabine Kleinert

**Affiliations:** 1 Sideview, Princes Risborough, UK; 2 System Analytic, London, UK; 3 The Lancet, London, UK

**Keywords:** Authorship, Misconduct, Researcher productivity, Publication ethics, Research integrity

## Abstract

**Introduction.** Researchers’ productivity is usually measured in terms of their publication output. A minimum number of publications is required for some medical qualifications and professional appointments. However, authoring an unfeasibly large number of publications might indicate disregard of authorship criteria or even fraud. We therefore examined publication patterns of highly prolific authors in 4 medical specialties.

**Methods.** We analysed Medline publications from 2008–12 using bespoke software to disambiguate individual authors focusing on 4 discrete topics (to further reduce the risk of combining publications from authors with the same name and affiliation). This enabled us to assess the number and type of publications per author per year.

**Results.** While 99% of authors were listed on fewer than 20 publications in the 5-year period, 24 authors in the chosen areas were listed on at least 25 publications in a single year (i.e., >1 publication per 10 working days). Types of publication by the prolific authors varied but included substantial numbers of original research papers (not simply editorials or letters).

**Conclusions.** Institutions and funders should be alert to unfeasibly prolific authors when measuring and creating incentives for researcher productivity.

## Introduction

The productivity of researchers is usually measured in terms of the number of publications they author. Some medical qualifications and academic appointments (e.g., ‘habilitation’ requirements in several countries) require a minimum number of publications in recognised, peer-reviewed journals (Buddeberg-Fischer, Stamm & Buddeberg, 2009). Press releases announcing institutional appointments often mention the appointee’s publication record. An informal web search produced examples mentioning that newly appointed individuals were already authors of 300, 400 or 1,000 peer-reviewed publications (E. Wager, pers. obs., data presented at 7th International Congress on Peer Review & Biomedical Publication, Chicago). One might therefore assume that the more publications a researcher is listed on, the better. However, authoring an unfeasibly large number of publications might suggest guest authorship or even fraud.

The link between extreme productivity and fraud is supported by anecdotal evidence. For example, the physicist Jan Hendrik Schön produced about 40 research papers in one year (submitting 7 in a single month), all of which were later retracted (Reich, 2009). Similarly, the discredited anaesthetist Yoshitaka Fujii published 30 clinical trials in a single year (Tramèr, 2013). The phenomenon of senior researchers abusing their positions and demanding guest authorship (i.e., listing despite making no, or minimal, contribution to the research) has also been documented (Kwok, 2005; Shulkin, Goin & Rennie, 1993).

We therefore examined researchers’ publication outputs to provide some initial insights into and measurements of the phenomenon of prolific authorship.

## Methods

Since simple searches of Medline by author name do not accurately identify individuals and cannot distinguish publications from different authors who have the same name, we used a bespoke, semi-automated tool (developed by SystemAnalytic) that considers additional author characteristics such as affiliation, publication history, and patterns of co-authorship. To further reduce the chance of combining publications from different authors with the same name, we focused on 4 discrete (arbitrarily chosen) topics (epilepsy, rheumatoid arthritis, renal transplantion and liver transplantation) which were defined by keywords in the Medline database. For each topic, we analysed all publications listed on Medline from January 2008 to December 2012. Using the software we characterized: the number of publications per individual, the types of publication, and patterns of author order. We also manually checked outputs for a convenience sample of the 10 most prolific authors for each topic using Medline to verify that these did, indeed, appear to be from single authors and to check the types of publication and authorship order.

## Results

We assessed 58,400 publications for 163,993 researchers. During the 5 years studied, 99% of researchers (162,744) were listed on fewer than 20 publications (Table 1). In contrast, the median total number of publications (excluding letters and editorials) for the most prolific authors was 93 (maximum 132, interquartile range 65–103). Considering individual years within this period, the maximum number of publications per year was 43 for any type of publication and 15 for clinical trials.

**Table 1:** Productivity of researchers in four selected topics indicated by the number of Medline publications 2008–12 per author and the maximum number for any one individual (Max).

Topic	Number of publications/author 2008-12 (*N*, %)					
	1–20	21–30	31–40	41–50	>50	Max
Epilepsy	63,866 (99.7)	141 (0.2)	34 (0.05)	11 (0.02)	37 (0.06)	118
Rheumatoid arthritis	33,953 (98.8)	124 (0.4)	66 (0.2)	30 (0.08)	41 (0.1)	149
Renal transplant	38,575 (99.1)	201 (0.5)	62 (0.2)	34 (0.1)	38 (0.1)	123
Liver transplant	26,350 (98.7)	174 (0.7)	69 (0.3)	36 (0.1)	56 (0.2)	128

Detailed, manual inspection and analysis of the output of the 40 most prolific authors (i.e., the top 10 for each topic) revealed great variations in the types of publications produced (Table 2). For example, one author published 32 letters and 22 review articles but only 1 primary research article reporting a clinical trial. Another author published 34 reports of clinical trials and 19 reviews but only 3 letters. The authors’ positions in the order of listing also varied, with several individuals featuring mainly as last author. The highest proportion of last author publications by an individual was 93/105 (89%), and the highest proportion of first author publications was 44/79 (56%). Of the 40 most prolific authors, 24 were listed on at least 25 publications in any single year (i.e., >1 publication per 10 working days).

**Table 2:** Types of publication and author order for the 10 most prolific authors in 4 medical specialties over 5 years (2008–12) on Medline.

Publication types										Author position (*N*(%)		
Auth. ID	Clin trial	Res art.	Case rep.	Editorial	Letter	Syst. rev.	Review	Total	Max/ year	1st	Middle	Last
1	61	37	7	7	11	6	20	149	38	16 (11)	79 (53)	54 (36)
2	26	62	4	1	13	7	25	138	43	1 (1)	106 (77)	31 (22)
3	34	72	4	3	3	3	19	138	35	6 (4)	77 (56)	55 (40)
4	6	83	25	0	8	0	6	128	31	3 (2)	47 (37)	78 (61)
5	46	45	5	2	18	1	7	124	27	0 (0)	103 (83)	21 (17)
6	10	66	19	4	13	0	8	120	31	17 (14)	101 (84)	2 (2)
7	26	62	12	2	7	0	10	119	25	1 (1)	64 (54)	54 (45)
8	1	56	3	4	32	0	22	118	30	0 (0)	70 (59)	48 (41)
9	7	82	9	1	3	2	9	113	33	5 (4)	79 (70)	29 (26)
10	41	20	29	0	10	1	11	112	27	7 (6)	46 (41)	59 (53)

**Notes.**

Auth ID author identifier (rank of top 10 most prolific authors) Clin trial report of a randomized clinical trial Res art research article Case rep case report Syst rev systematic review

The 40 most prolific authors were based in 5 continents (none from Africa) and a range of countries, the most common being Germany (6), the Netherlands (6), and Japan (4). We did not find any Medline retractions associated with these authors.

## Discussion and Conclusions

Using specially designed software, we were able to calculate the number of publications per individual researcher and thus provide some measures of prolific authorship in a range of medical fields. More detailed analysis also revealed the publication patterns of the most prolific authors and we found these to be highly variable.

Judging by our manual analysis of the 40 most prolific authors, the software (originally designed to analyse publication patterns of medical opinion leaders) successfully identified the output of individual researchers. We limited our search to Medline and did not consider other publications such as conference abstracts or those in non-listed journals. This means our estimates of prolific output are conservative and may not be generalizable to other disciplines. This was an exploratory, observational study to give an initial impression on the phenomenon of high author productivity. We did not attempt to assess the causes for high productivity.

Since our initial study, another group has examined authorship patterns among diabetes researchers and noted that the most prolific were named, on average, on 7 articles reporting clinical trials per year, for 10 years (Holleman et al., 2015). One limitation both of our study and that by Holleman et al. is that neither attempted to determine the number of trials described in these publications, so one explanation of some of the apparently prolific authors is that they were involved mainly with very large studies that generated many publications (Wager, 2015). However, the work required to take part in both the research and publication of such large trials is still substantial and such high productivity raises questions, at the very least, about how authorship guidelines are being interpreted.

While special techniques are currently required to analyse the output of all biomedical researchers, and thus get information about normal and abnormal productivity, it is straightforward to assess the output of a small number of individuals and to validate this manually. We therefore suggest that institutions and funders should be alert to the possibility of excessive authorship. One simple technique would be to require job or research funding applicants to include a total publication count in their application or CV. Spotting or verifying over-prolific authors should become easier in future if journals and databases adopt researcher identification systems such as ORCID (http://orcid.org) rather than relying simply on author names for identification. Although the absolute number of highly prolific authors in each field is probably small, asking researchers to justify their authorship, if there are any suspicions, shows that institutions take research integrity seriously. Abusive authorship patterns, such as senior figures who demand to be listed on publications despite having had little or no involvement in research are well documented (Kwok, 2005) and can have damaging effects on junior researchers because they send a signal that honest authorship is unimportant.

We suggest that institutional authorship policies and guidelines should stress the importance of following accepted authorship criteria (which may differ between disciplines) and that institutions should have systems in place to handle suspected abuses. Many guidelines note that authorship entails accountability for the research being reported (ICMJE, 2013; Anonymous, 2007) and this aspect should be reflected in policies and training. Institutions should also consider how to reduce the ‘publish or perish’ atmosphere, often cited as a factor in misconduct and questionable research practices, and how to create an environment that encourages integrity and honest authorship practices (Wager, 2015). Appointment and tenure committees should also develop methods to measure the quality rather than merely the quantity of a researcher’s publications.

## Additional Information and Declarations

### Data Availability

The following information was supplied regarding the deposition of related data: Anonymised data are available from the corresponding author (Dr. Elizabeth Wager, liz@sideview.demon.co.uk) on request and will be shared with other researchers for appropriate projects on the understanding that the identity of individuals will not be disclosed.
